# The value of the apoB/apoAΙ ratio and the non-HDL-C/HDL-C ratio in predicting carotid atherosclerosis among Chinese individuals with metabolic syndrome: a cross-sectional study

**DOI:** 10.1186/s12944-015-0023-4

**Published:** 2015-04-09

**Authors:** Guangming Qin, Jiangfeng Tu, Chenjing Zhang, Xiaoxiao Tang, Laisheng Luo, Jiaqi Wu, Lingang Liu, Wen Lu, Lisha Tao, Shengrong Shen, Undurti N Das, Wensheng Pan

**Affiliations:** Department of Laboratory, The Second Affiliated Hospital, School of Medicine, Zhejiang University, Hangzhou, 310009 China; Department of Gastroenterology, The Second Affiliated Hospital, School of Medicine, Zhejiang University, 88# Jiefang Road, Hangzhou, 310009 China; School of Biosystems Engineering and Food Science, Zhejiang University, Hangzhou, 310029 China; UND Life Sciences, 2020 S 360th St., Federal Way, WA 98003 USA; Department of Gastroenterology, The Second Affiliated Hospital Binjiang Campus, School of Medicine, Zhejiang University, Hangzhou, 310009 China

**Keywords:** Carotid atherosclerosis, Carotid intima-media thickness, Metabolic syndrome, ApoB/apoAΙ, Non-HDL-C/HDL-C

## Abstract

**Background:**

Metabolic syndrome (MetS) is associated with carotid intima-media thickness (CIMT), which is a good predictor of cardiovascular disease (CVD). However, among individuals with MetS, direct comparative data regarding the utility of the apoB/apoAΙ ratio and the non-HDL-C/HDL-C ratio to diagnose carotid atherosclerosis are scarce, particularly in Chinese populations. We aimed to determine the relationship between the apoB/apoAΙ ratio and the non-HDL-C/HDL-C ratio and carotid atherosclerosis among Chinese individuals with MetS.

**Methods:**

We performed a retrospective study of 5822 Chinese participants who underwent a routine health screening examination. Lipid profiles, fasting glucose, fasting insulin, CRP, apoB, apoAΙ and CIMT were measured.

**Results:**

We observed that among Chinese individuals with MetS, men (53.95 ± 0.58 ys) developed carotid atherosclerosis at a younger age than women (58.47 ± 1.17 ys) (*P* < 0.001). Both the apoB/apoAΙ ratio and the non-HDL-C/HDL-C ratio positively correlated with carotid atherosclerosis among Chinese individuals with MetS, particularly among women. Meanwhile, CIMT increased progressively across the quartiles of the non-HDL-C/HDL-C ratio (*P* for trend, < 0.05). Receiver Operating Characteristic (ROC) analysis indicated that the AUC of the apoB/apoAΙ ratio (0.561) was higher than that of the non-HDL-C/HDL-C ratio (0.522) in men (*P* < 0.05) and the AUC of the apoB/apoAΙ ratio (0.640) was lower than that of the non-HDL-C/HDL-C ratio (0.695) in women (*P* < 0.05). Among Chinese individuals with MetS, the AUC of the non-HDL-C/HDL-C ratio was more prominent among women compared with men (*P* < 0.05).

**Conclusion:**

Our findings indicate that among individuals with MetS, Chinese men develop carotid atherosclerosis at a much younger age than women. There were no significant differences between the apoB/apoAΙ ratio and the non-HDL-C/HDL-C ratio for the prediction of carotid atherosclerosis among Chinese individuals with MetS. Among Chinese individuals with MetS, the utility of the non-HDL-C/HDL-C ratio was found to be greater among women than among men.

## Introduction

Metabolic syndrome (MetS) is strongly associated with various cardiovascular risk factors, including visceral obesity, hypertension, hypertriglyceridemia, a low level of high density lipoprotein cholesterol, and impaired glucose tolerance [1,2]. Individuals with MetS are at an increased risk of developing cardiovascular disease, cardiovascular morbidity, mortality, and all-cause mortality [3,4]. The prevalence is estimated to be approximately 34% among adults in the USA [5], and 14%-17% among adults in China [6]. These rates are likely to increase due to the aging of the population.

Carotid intima-media thickness (CIMT) is an early indicator of atherosclerosis [7,8], and is a validated measurement of subclinical atherosclerosis [9]. Among the indicators of cardiovascular disease, CIMT is considered to be an early marker of the disease; therefore, it is widely used as a diagnostic tool [10,11]. Several studies have used both the apolipoprotein B (apoB)/apolipoprotein AΙ (apoAΙ) ratio and the non-HDL-C/HDL-C ratio to estimate the likelihood of having carotid atherosclerosis. In the setting of both type 2 diabetes mellitus and normal glucose tolerance (NGT), the apoB/apoAΙ ratio is more strongly associated with CIMT than conventional lipids [12,13]. Generally, the non-HDL-C/HDL-C ratio is believed to be superior to traditional lipid variables in estimating arterial stiffness among middle-aged and elderly Chinese individuals [14].

Several studies have described the relationship between MetS and CIMT [3,15-17]. However, direct comparative data regarding the utility of the apoB/apoAΙ ratio, the non-HDL-C/HDL-C ratio, insulin resistance (IR), conventional lipids and the relationship between these parameters and increased CIMT are scarce among individuals with MetS. Given the increasing incidence of MetS in China, it is important to identify the best method with which to assess the risk of cardiovascular disease (CVD). Very few studies have assessed the utility of apolipoprotein and lipoprotein cholesterol measurements in determining the risk of CVD among Chinese individuals. In the present study, we examined the relationship among carotid atherosclerosis, the apoB/apoAΙ ratio, and the non-HDL-C/HDL-C ratio, as well as conventional lipids, IR, and C reactive protein (CRP), among Chinese individuals with MetS. We also aimed to determine whether gender differences exist in this association [18].

## Methods

### Subjects

We collected data from 5822 subjects between 18 and 85 years of age who underwent a health examination at the Second Affiliated Hospital, School of Medicine, Zhejiang University, between September 2011 and December 2012. Most of the patients were from Zhejiang province. 4908 individuals were included in the statistical analysis, of whom 3039 men, aged 45.12 ± 9.10 years, and 1869 women, aged 44.92 ± 9.79 years (*P* > 0.05) were ultimately included in the study, following the exclusion of individuals with either cardiovascular or cerebrovascular disease, cancer, diabetes, severe mental disorders, or chronic kidney disease [19], as well as individuals receiving lipid-lowering agents, anti-thyroid agents, and individuals with acute and chronic liver disease, infectious diseases, acute and chronic fevers of unknown origin, and who consumed excessive alcohol [20]. Among these individuals, 1863 were diagnosed with MetS, 1492 of which were men, with a mean age of 46.80 ± 8.83 years, and 371 were women, with a mean age of 51.09 ± 9.39 years (*P* < 0.001). This study was reviewed and approved by the institutional review board of the Second Affiliated Hospital of Zhejiang University School of Medicine (ethical review code: Research 2014–113). All subjects provided written informed consent.

### The definition of metabolic syndrome

In our study, metabolic syndrome was defined by the presence of 3 or more of the following conditions, based on the NECP ATP III criteria [21]: 1) waist circumference (WC) ≥ 90 cm in men and ≥ 80 cm in women, 2) triglycerides ≥ 150 mg/dL, 3) HDL cholesterol < 40 mg/dL in men and < 50 mg/dL in women, 4) blood pressure ≥ 130/85 mm Hg or receiving antihypertensive medication, and 5) fasting glucose ≥ 100 mg/dL.

### Questionnaire and physical examination

A standardised questionnaire was generated by a physician to collect baseline information such as age, gender, smoking habits and drinking habits. A physical examination was performed by a physician to collect information such as weight, height and waist circumstance. The body mass index was calculated as the weight in kilograms divided by the height in meters squared. Both systolic and diastolic blood pressure were measured in the right following 5 minutes of rest, with the patient in a sitting position, using an automated device (Omron 711, USA), and the mean of two consecutive blood pressure measurements was recorded.

### Laboratory tests

All subjects fasted for 12 hours, and their venous blood samples were collected for the measurement of fasting plasma glucose (FPG), blood profiles (total cholesterol: TC, triglyceride: TG, high density lipoprotein–cholesterol: HDL-C, low density lipoprotein- cholesterol: LDL-C, apolipoprotein B: apoB, apolipoprotein AΙ: apoAΙ), homocysteine (HCY), gamma-glutamyl transpeptidase (GGT), uric acid (UA), fasting insulin (FINS), and C-reactive protein (CRP). FPG was measured via the hexokinase (HK) method; TC was measured via the cholesterol oxidase-peroxidase method (CHO-POD); TG was measured via the glycerol phosphate oxidase-peroxidase method; HDL-C was measured via the direct method-surfactant clearance method; LDL-C was measured via the direct method-selected inhibitor method; HCY was measured using an enzymatic cycling assay; GGT was measured via the modified SZASZ method; UA was measured via the urease-peroxidase method; ApoB, apoAΙ, and CRP were each measured using a turbidimetric immunoassay with a BECKMAN COULTER AU5400 Analyzer. FINS was measured via direct chemical luminescence using with a SIEMENS ADVIA CENTAUR XP Analyzer. The homeostasis model assessment of insulin resistance (HOMA-IR) was calculated based on the HOMA model, as follows: HOMA-IR = fasting glucose (mmol/L) * fasting plasma insulin (mU/L)/22.5.

To guarantee both the accuracy and the comparability of the results, two quality control materials (products of BIO-RAD, lot numbers of liquid assayed multiqual: 45641 and 45643, lipids control: 57251 and 57252) of different concentrations were tested each day. The cumulative CVs were as follows: FPG (1.83%; 1.73%), TG (4.0%; 2.56%), TC (2.05%; 1.92%), HDL-C (2.60%; 2.70%), LDL-C (3.03%; 3.01%), apoB (<10%; <10%), apoAΙ (<10%; <10%), GGT (<6%; <4%), HCY (<6%; <6%), UA (<3%; <3%), FINS (<10%; <10%), and CRP (<10%; <10%).

### Carotid ultrasound measurement

CIMT measurements were performed by an experienced sonographer, using a high-resolution B-mode tomographic ultra-sound system (GE LOGIQ E9, USA), with a linear 9 MHz transducer. The distal segment and stigma compartments of the cephalic artery and the proximal segment of the internal carotid artery were measured on both sides [22]. The transducer was manipulated so that the luminal diameter was maximised in the longitudinal plane. Each of the above blood vessels was measured in three different sections, within a range of 1 cm in the proximal wall and distant from the sidewalls. Carotid atherosclerosis was diagnosed when CIMT was ≥ 0.9 mm [23,24].

### Statistical analysis

All statistical analyses were performed using SPSS 17 software. Data were expressed either as means ± S.D. or as geometrical means (95% confidence intervals [CIs]) for the continuous variables, and as percentages for the categorical variables. TG was analysed after log-transformation due to a skewed distribution. General characteristics were compared separately among participants with and without carotid atherosclerosis, in both men and women with MetS, using the Student’s *t*-test. The categorical variables were analysed via the Chi-square test. Spearman correlation coefficients were used to study the relationships between the different risk factors and carotid atherosclerosis. The relationships between the different lipid ratios and carotid atherosclerosis were each assessed using binary logistic regression analysis with forward selection method and multinomial logistic regression analysis. Odds ratios (ORs) and 95% confidence intervals (CIs) for carotid atherosclerosis were each calculated. Regarding the multinomial logistic regression analysis, age, body mass index (BMI), systolic blood pressure (SBP), diastolic blood pressure (DBP), TC, TG, HDL-C, LDL-C, apoB, apoAΙ, INS, IR, CRP, HCY, GGT, and UA were included as covariates. To assess the utility of the different lipid ratios as markers of MetS, we constructed sex-specific Receiver Operating Characteristic (ROC) curves and compared the areas under the ROC curves (AUCs) with the Z-statistic. A *P* value less than 0.05 was considered to be statistically significant.

## Results

### The relationship between MetS and carotid atherosclerosis

A total of 4908 subjects were included in the analysis. The sample consisted of 3039 men and 1869 women. The age range was 20–84 years in men and 20–83 years in women. Based on the NECP ATP III criteria, 1863 people were diagnosed with MetS, which was present in 49.1% (1492/3039) of men and 19.9% (371/1869) of women. Among Chinese individuals with MetS, 16.6% (309/1863) of the individuals had carotid atherosclerosis, including 16.6% (247/1492) of men and 16.7% (62/371) of women. Among Chinese individuals without MetS, 9.5% (289/3045) of the individuals had carotid atherosclerosis. MetS remained significantly associated with carotid atherosclerosis (OR 1.90, 95% CI 1.58–2.25, *P* < 0.001). Therefore, MetS is a significant risk factor for carotid atherosclerosis.

### The clinical and biochemical characteristics of the participants with and without carotid atherosclerosis among Chinese individuals with MetS

Among Chinese individuals with MetS, the clinical and biochemical characteristics including the metabolic components and the lipid profiles of both men and women with and without carotid atherosclerosis are shown in Table 1. The characteristics are stratified by the presence of carotid atherosclerosis. The mean apoB/apoAΙ ratios were significantly higher among the participants with carotid atherosclerosis (0.91 ± 0.02 in men and 0.85 ± 0.03 in women) compared to participants without carotid atherosclerosis (0.85 ± 0.01 in men and 0.75 ± 0.01 in women) (*P* < 0.05). Additionally, there were significant differences in the TC, LDL-C, FINS, SBP, DBP, UA and apoB levels between the two groups (*P* < 0.05). There were also significant differences in the HOMA-IR and FPG levels among men, and the TG and the non-HDL-C/HDL-C ratio levels among women. The men (53.95 ± 0.58 ys) developed carotid atherosclerosis at a younger age than the women (58.47 ± 1.17 ys), among Chinese individuals with MetS (*P* < 0.001).

**Table 1:** **Clinical and biochemical characteristics in cluding metabolic components and lipid measures in participant with and without the carotid atherosclerosis in both genders**

**Variables**	**Men**	**Women**				
	**Carotid atherosclerosis (−); n = 1245**	**Carotid atherosclerosis (+); n = 247**	***P*** **-value**	**Carotid atherosclerosis (−); n = 309**	**Carotid atherosclerosis (+); n = 62**	***P*** **-value**
Age (ys)	45.14 ± 0.23	53.95 ± 0.58	<0.001	49.61 ± 0.50	58.47 ± 1.17	<0.001
SBP (mm Hg)	137.83 ± 0.31	142.69 ± 1.01	<0.001	137.28 ± 0.61	142.52 ± 1.55	<0.001
DBP (mm Hg)	80.10 ± 0.27	90.16 ± 0.73	<0.001	86.40 ± 0.53	88.53 ± 1.39	<0.001
BMI (kg/m^2^)	25.75 ± 0.08	25.63 ± 0.17	0.544	24.26 ± 0.17	25.23 ± 0.39	0.021
FPG (mmol/L)	5.70 ± 0.03	5.96 ± 0.10	0.016	5.62 ± 0.06	5.65 ± 0.09	0.848
FINS (pmol/L)	70.01 ± 1.10	60.49 ± 2.21	<0.001	62.99 ± 1.75	72.06 ± 4.63	0.041
HOMA-IR	2.58 ± 0.05	2.34 ± 0.11	0.034	2.28 ± 0.07	2.62 ± 0.18	0.056
TC (mmol/L)	5.28 ± 0.03	5.46 ± 0.06	0.007	5.38 ± 0.05	5.74 ± 0.12	0.005
HDL-C (mmol/L)	1.36 ± 0.01	1.38 ± 0.02	0.284	1.64 ± 0.02	1.53 ± 0.04	0.01
non-HDL-C/HDL-C	2.97 ± 0.02	3.06 ± 0.06	0.149	2.37 ± 0.04	2.82 ± 0.08	<0.001
LDL-C (mmol/L)	3.44 ± 0.02	3.55 ± 0.05	0.041	3.29 ± 0.04	3.65 ± 0.09	0.001
TG (mmol/L)	2.31 ± 0.04	2.13 ± 0.08	0.054	1.72 ± 0.04	2.14 ± 0.13	<0.001
ApoAΙ (g/L)	1.38 ± 0.01	1.40 ± 0.01	0.321	1.54 ± 0.01	1.51 ± 0.02	0.26
ApoB (g/L)	1.17 ± 0.01	1.25 ± 0.02	<0.001	1.13 ± 0.02	1.27 ± 0.04	0.001
ApoB/apoAΙ	0.85 ± 0.01	0.91 ± 0.02	0.001	0.75 ± 0.01	0.85 ± 0.03	0.001
CRP (mg/L)	5.35 ± 0.12	5.67 ± 0.48	0.363	5.34 ± 0.21	4.57 ± 0.35	0.117
HCY (μmol/L)	11.16 ± 0.15	11.6 ± 0.29	0.186	8.28 ± 0.18	9.76 ± 0.38	0.001
GGT (U/L)	61.54 ± 1.60	54.47 ± 3.72	0.074	23.69 ± 1.13	30.95 ± 3.86	0.075
UA (μmol/L)	371.29 ± 2.15	360.94 ± 4.51	0.048	257.46 ± 3.07	283.81 ± 7.56	0.001
Current smoker(%)	41.5	39.7	>0.05	0.6	0	>0.05

SBP: systolic blood pressure; DBP: diastolic blood pressure; BMI: body mass index; FPG: fasting plasma glucose; FINS: fasting insulin; HOMA-IR: homeostasis model assessment of insulin resistance; TC: total cholesterol; HDL-C: high density lipoprotein cholesterol; LDL-C: low density lipoprotein cholesterol; non-HDL-C: non-high density lipoprotein cholesterol; TG: triglycerides; ApoAΙ: apolipoprotein AΙ; ApoB: apolipoprotein B; CRP: C-reactive protein; HCY: Homocysteine; GGT: gamma-glutamyl transpeptidase; UA: uric acid; Carotid atherosclerosis(+): CIMT was ≥ 0.9 mm; Carotid atherosclerosis(−): CIMT was < 0.9 mm.

### Relationship between carotid atherosclerosis and blood lipid profile and ratios

Spearman correlations between carotid atherosclerosis and the apoB/apoAΙ ratio and the non-HDL-C/HDL-C ratio are shown in Table 2. Both the apoB/apoAΙ ratio and the non-HDL-C/HDL-C ratio positively correlated with carotid atherosclerosis. The Spearman correlation coefficients between carotid atherosclerosis and the apoB/apoAΙ ratio and the non-HDL-C/HDL-C ratio were higher in women compared to men. Binary logistic regression analysis with forward selection method was used to assess the OR for CIMT, as shown in Table 3. Among men, the apoB/apoAΙ ratio was more prominent than age, SBP, and TG. Among women, the non-HDL-C/HDL-C ratio was more prominent than age.

**Table 2:** **Spearman correlations between carotid atherosclerosis and risk factors for atherosclerosis in both genders**

**Variables**	**Men**	**Women**		
	**Correlation coefficient**	***P*** **-value**	**Correlation coefficient**	***P*** **-value**
ApoB/apoAΙ	0.079	0.002	0.181	<0.001
Non-HDL-C/HDL-C	0.067	0.01	0.2	<0.001
HDL-C (mmol/L)	0.017	0.508	−0.151	0.004
LDL-C (mmol/L)	0.052	0.044	0.181	<0.001
TC (mmol/L)	0.07	0.007	0.141	0.006
ApoB (g/L)	0.103	<0.001	0.162	0.002
ApoAΙ (g/L)	0.018	0.485	−0.06	0.25
HOMA-IR	−0.082	0.002	0.102	0.05
CRP (mg/L)	−0.017	0.514	−0.069	0.187
Current smoker	−0.014	0.59	−0.033	0.527

HDL-C: high density lipoprotein cholesterol; non-HDL-C: non-high density lipoprotein cholesterol; LDL-C: low density lipoprotein cholesterol; TC: total cholesterol; ApoB: apolipoprotein B; ApoAΙ: apolipoprotein AΙ; HOMA-IR: homeostasis model assessment of insulin resistance; CRP: C-reactive protein.

**Table 3:** **Odds ratio and 95% confidence interval of carotid atherosclerosis associated with blood lipid profile or ratio in both genders**

	**Variables**	**OR**	**95% CI**	***P*** **-value**
men				
	apoB/apoAΙ	6.142	3.219-11.720	<0.001
	Age (ys)	1.129	1.107-1.151	<0.001
	SBP (mm Hg)	1.014	1.003-1.026	0.015
	TG (mmol/L)	0.671	0.475-0.949	0.024
women				
	non-HDL-C/HDL-C	2.054	1.338-3.153	0.001
	Age (ys)	1.112	1.072-1.153	<0.001

ApoB: apolipoprotein B; ApoAΙ: apolipoprotein AΙ; SBP: systolic blood pressure; TG: triglyceride; HDL-C: high density lipoprotein cholesterol; non-HDL-C: non-high density lipoprotein cholesterol.

### The risk of carotid atherosclerosis based on the apoB/apoAΙ ratio and the non-HDL-C/HDL-C ratio quartiles in each sex among Chinese individuals with MetS

Following an adjustment for age, BMI, SBP, DBP, TC, TG, HDL-C, LDL-C, apoB, apoAΙ, INS, IR, CRP, HCY, GGT, and UA, the ORs of the two ratios increased significantly in both men and women, as shown in Table 4. As compared with quartile 1 (reference), quartile 2 (OR = 1.205), quartile 3 (OR = 1.541) and quartile 4 (OR = 2.465) of the apoB/apoAΙ ratio in men and quartile 2 (OR = 1.587), quartile 3 (OR = 2.034) and quartile 4 (OR = 2.772) of the apoB/apoAΙ ratio in women were significantly associated with carotid atherosclerosis. Similar relationships were observed for the non-HDL-C/HDL-C ratio, with the same adjusted, ORs for the increasing quartiles of the non-HDL-C/HDL-C ratio of quartile 1 (reference), 1.287, 1.627 and 1.843 in men, and quartile 1 (reference), 1.383, 3.200 and 5.847 in women. The ORs were significantly greater in the highest apoB/apoAΙ ratio quartile compared with the lowest apoB/apoAΙ ratio quartile, as well as the non-HDL-C/HDL-C ratio, in both men and women (*P* < 0.05). Meanwhile, CIMT increased progressively across the quartiles of the non-HDL-C/HDL-C ratio (*P* for trend, < 0.05). The increase in ORs was more prominent among women than among men.

**Table 4:** **The risk of carotid atherosclerosis according to quartiles of apoB/apoAΙ ratio and non-HDL-C/HDL-C ratio in both genders**

		**Ratio levels** ^*^				***P*** **for trend**
		**Q1**	**Q2**	**Q3**	**Q4**	
Men						
	range of apoB/apoAΙ	<0.690	0.690-0.841	0.841-1.000	>1.000	
	ORs for carotid atherosclerosis	1	1.205(0.768-1.890)	1.541(1.003-2.336)	2.465(1.598-3.803)	<0.001
	range of non-HDL-C/HDL-C	<2.390	2.390-2.937	2.937-3.508	>3.508	
	ORs for carotid atherosclerosis	1	1.287(0.834–1.987)	1.627(1.060-2.499)	1.843(1.189-2.856)	0.177
Women						
	range of apoB/apoAΙ	<0.594	0.594-0.764	0.764-0.888	>0.888	
	ORs for carotid atherosclerosis	1	1.587(0.573-4.392)	2.034(0.758-5.496)	2.772(1.051-7.315)	<0.001
	range of non-HDL-C/HDL-C	<1.885	1.885-2.393	2.393-2.955	>2.955	
	ORs for carotid atherosclerosis	1	1.383(0.424–4.511)	3.200(1.077-9.511)	5.847(1.982-17.245)	0.001

^*^Covariates for adjustment including age, BMI, SBP, DBP, TC, TG, HDL-C, LDL-C, apoB, apoAΙ, INS, IR, CRP, HCY, GGT, and UA. ApoB: apolipoprotein B; ApoAΙ: apolipoprotein AΙ; HDL-C: high density lipoprotein cholesterol; non-HDL-C: non-high density lipoprotein cholesterol.

### The diagnostic values of different lipid ratios in detecting carotid atherosclerosis among Chinese individuals with MetS

**Figure 1:**
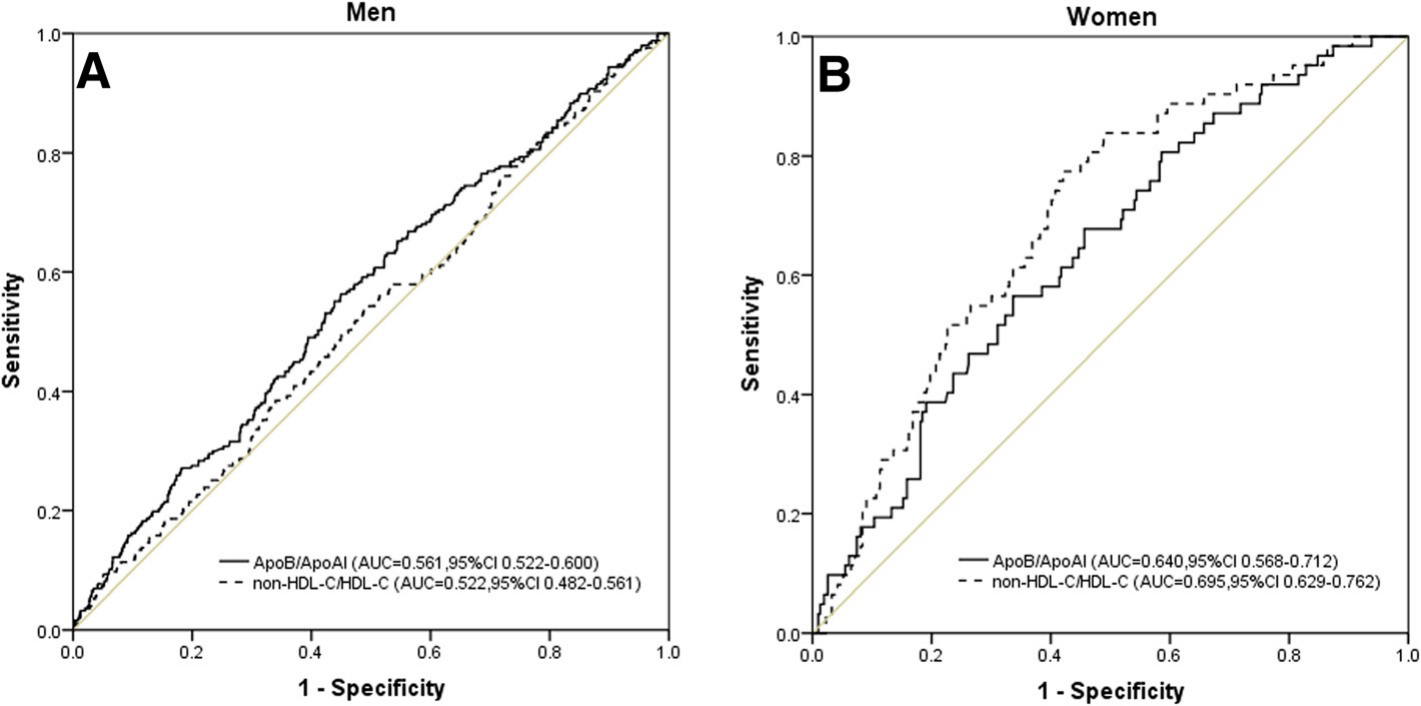
**Discriminatory power of apoB/apoAΙ and non-HDL-C/HDL-C for carotid atherosclerosis by Receiver Operating Characteristic (ROC) curves in men (A) and women (B)** ApoB: apolipoprotein B; ApoAΙ: xapolipoprotein AΙ; HDL-C: high density lipoprotein cholesterol; non-HDL-C: non-high density lipoprotein cholesterol.

To compare the predictive values of the different ratios in predicting carotid atherosclerosis, we analysed the ROC curves of the two ratios (apoB/apoAΙ and non-HDL-C/HDL-C). ROC analysis indicated that the AUC of the apoB/apoAΙ ratio (0.561) was higher than that of the non-HDL-C/HDL-C ratio (0.522) in men (*P* < 0.05) and the AUC of the apoB/apoAΙ ratio (0.640) was lower than that of the non-HDL-C/HDL-C ratio (0.695) in women (*P* < 0.05). Graphical comparisons of the ROC curves of these ratios relative to carotid atherosclerosis are presented in Figure 1. Figures A and B depicted the discriminatory value of these two ratios relative to carotid atherosclerosis in men and women, respectively. There was no significant difference between the apoB/apoAΙ ratio and the non-HDL-C/HDL-C ratio regarding the AUC with the Z-statistic (*P* > 0.05). The AUC of the apoB/apoAΙ ratio was not larger in women than in men (*P* > 0.05), whereas the AUC of the non-HDL-C/HDL-C ratio was larger in women than men (*P* < 0.05). The AUCs for the other parameters were as follows: TG (AUC = 0.457 in men, AUC = 0.637 in women), non-HDL-C (AUC = 0.552 in men, AUC = 0.654 in women), HDL-C (AUC = 0.513 in men, AUC = 0.383 in women), TC (AUC = 0.555 in men, AUC = 0.609 in women), LDL-C (AUC = 0.540 in men, AUC = 0.640 in women), IR (AUC = 0.436 in men, AUC = 0.579 in women), CRP (AUC = 0.487 in men, AUC = 0.447 in women), apoB (AUC = 0.580 in men, AUC = 0.626 in women), and apoAΙ (AUC = 0.514 in men, AUC = 0.454 in women).

## Discussion

Previous studies have shown that patients with MetS are at increased risk of developing vascular diseases, including endothelial dysfunction [3,4,25,26]. The Botnia Study demonstrated that the risk of both coronary heart disease and stroke increased three-fold among subjects with metabolic syndrome [4]. In a retrospective analysis of 5822 men and women, we observed that the risk of carotid atherosclerosis in a Chinese population with MetS was approximately twice that of individuals without MetS (*P* < 0.001).

In the present study, men were found to be at a higher risk for carotid atherosclerosis compared with women. Among patients with carotid atherosclerosis, men were found to be younger than women, which may be the result of a lower level of apoB, as apoB protects against the development of atherosclerosis, and is a good predictor of CVD [13,27-29]. The anti-atherosclerotic action of oestrogen may also play a role in this process [30]. Based on these results, we believe that men with Mets should be screened for CIMT at a much younger age compared with women.

Several studies have demonstrated that the apoB/apoAΙ ratio and the non-HDL-C/HDL-C ratio may be used to predict the risk of carotid atherosclerosis [12-14]. Chiadi E Ndumele reported that there were no significant differences between the ability of the apoB/apoAΙ ratio and the ability of the non-HDL-C/HDL-C ratio to predict coronary heart disease in the setting of MetS [31]. However, none of these previous studies compared the ratio of apoB/apoAΙ with the ratio of non-HDL-C/HDL-C relative to carotid atherosclerosis among Chinese individuals with MetS. Therefore, in addition to the correlation between these two ratios and carotid atherosclerosis among Chinese individuals with MetS, we compared their discriminatory values relative to carotid atherosclerosis among Chinese individuals with MetS. We noted that the ratios of apoB/apoAΙ and non-HDL-C/HDL-C each positively correlated with carotid atherosclerosis among Chinese individuals with MetS, particularly women. The results of the ROC analysis indicated that there were no significant differences in the abilities of the ratios of apoB/apoAΙ and non-HDL-C/HDL-C to predict carotid atherosclerosis among Chinese individuals with MetS.

The AUC of the non-HDL-C/HDL-C ratio was significantly lower among men than among women, suggesting that women were more sensitive and relatively more susceptible to the burden of this ratio compared with men. This phenomenon was also observed in both white and black individuals [32]. The exact reason for this result must be determined in future studies. This phenomenon may result from changes in the levels of both HDL-C and TG, which have been found to exert stronger effects among women [33].

This study had several limitations. The number of women subjects was small; therefore, the observed effects may not be applicable to the general population. As this was a retrospective study, medication histories (except lipid-lowering agents), histories of previous medical or surgical diseases, durations of diabetes, or possible intolerance to medications may have been additional limiting factors in the extrapolation of the present findings to the general population. Given the cross-sectional nature of our study, the impact of these two ratios and their interaction with MetS components on cardiovascular outcomes could not be ascertained. Hence, we hope to learn the impacts of the comparisons and the diagnostic values of the investigated ratios in detecting cardiovascular events or diabetes in a future study. Despite these limitations, the non-HDL-C/HDL-C ratio and the apoB/apoAΙ ratio may each be useful in predicting cardiovascular events.

## Conclusion

Our findings indicate that Chinese men with MetS develop carotid atherosclerosis at a much younger age than women with MetS. There were no significant differences in the ability to predict the risk of carotid atherosclerosis between the apoB/apoAΙ ratio and the non-HDL-C/HDL-C ratio among Chinese individuals with MetS. Among Chinese individuals with MetS, the utility of the non-HDL-C/HDL-C ratio was found to be greater among women than among men.
